# Supplementary material for: Potential Lifshitz transition at optimal substitution in nematic pnictide Ba1−xSrxNi2As2
Source: Sci Adv. 2023 Oct 18;9(42):eadi4966. doi: 10.1126/sciadv.adi4966 (PMC10584352; doi:10.1126/sciadv.adi4966)
Supplement: Supplementary file 1 — Supplementary Materials Figs. S1 to S7 Table S1 [file sciadv.adi4966_sm.pdf]

Science Advances
AAAS

Supplementary Materials for

Potential Lifshitz transition at optimal substitution in nematic pnictide $Ba_{1-x}Sr_xNi_2As_2$

Dushyant M. Narayan *et al.*

Corresponding author: Dushyant M. Narayan, duna1846@colorado.edu;
Daniel S. Dessau, dan.dessau@colorado.edu

Sci. Adv. **9**, eadi4966 (2023)
DOI: 10.1126/sciadv.adi4966

This PDF file includes:

Supplementary Materials
Figs. S1 to S7
Table S1

Supplementary Materials

$\mathbf{k_z}$ **and photon energy selection**: To determine the correct photon energies to use for ARPES studies of $Ba_{1-x}Sr_xNi_2As_2$, we undertook photon energy scans of several compounds throughout the substitution range, and compared them with their respective DFT calculations. In figure S1, we can see the resulting Fermi-surface maps in the k_x - k_z plane with the corresponding overlays from DFT.

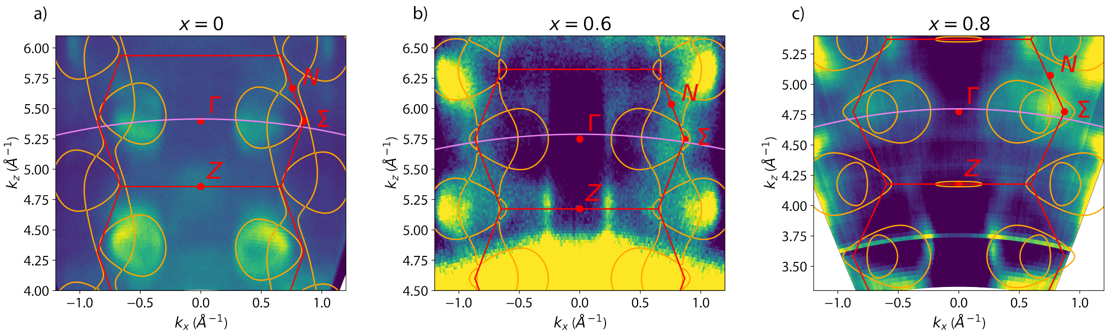

Figure S1: **Fermi-surface maps along $\mathbf{k_x - k_z}$ plane extracted from photon energy scans with DFT overlays (orange). a)** $x = 0$ parent compound photon energy scan taken in the triclinic phase at $T = 12$K with LH polarization from 50 to 150 eV. Inner potential was determined to be 16 eV. Violet line corresponds to 100 eV, where the rest of the data was taken for this substitution level. Overlay from DFT calculation of triclinic parent compound. **b)** $x = 0.6$ compound photon energy scan taken in the tetragonal phase at $T = 200$K with LV polarization from 65 to 160 eV. Inner potential was determined to be 16 eV. Violet line corresponds to 115 eV, where the rest of the data for this substitution level was taken. Overlay from DFT calculation of tetragonal phase using structural data extracted from XRD refinement of $x = 0.6$ crystals. **c)** $x = 0.8$ compound photon energy scan taken in the tetragonal phase at $T = 200$K with LV polarization from 30 to 120 eV. Inner potential was determined to be 17 eV. Violet line corresponds to 75 eV, where the rest of the data for this substitution level was taken. Like **b)**, overlay for **c)** also taken from tetragonal structure DFT calculation using structural data extracted from XRD refinement of $x = 0.8$ crystals.

The resulting photon energy scans were k-converted using inner potentials of 16, 16 and 17 eV for $x = 0$, $x = 0.6$ and $x = 0.8$ respectively, and show good agreement between the ARPES experimental data and DFT. Extra states can be seen in the $x = 0.6$ and $x = 0.8$ compounds

that are non-dispersive and not captured by the DFT (vertical lines in the data on either side of Γ and Z points). Due to the lack of k_z dispersion, which indicates confinement of the states along the real-space z direction, these are likely surface states.

By performing the photon energy scans, and determining the inner potential for each compound, we can now determine the photon energies we must use to measure in-plane ARPES FS maps and cuts at the correct k_z values. In figure S2, we can see the photon energies and k_z values for the Γ and Z points as a function of Sr-substitution.

Using the experimental lattice parameters as extracted from XRD, we can calculate the associated reciprocal lattice vectors and distances in k space between each successive Γ and Z point. Using these calculations and the inner potentials for each compound, we can create a map of the k_z values vs. Sr substitution which leads to figure S2. In figure S2, we can see a smooth evolution of k_z values for the high symmetry planes, and the associated photon energies needed to reach them. This is consistent with the smooth evolution of lattice parameters in the $Ba_{1-x}Sr_xNi_2As_2$ system through the substitution range. Using this data, we identify 100 eV, 115 eV, and 75 eV as photon energies to use to reach the Γ plane in k_z for the $x = 0$, $x = 0.6$ and $x = 0.8$ compounds respectively. With these photon energies in hand, we can now probe the in-plane electronic structure of $Ba_{1-x}Sr_xNi_2As_2$

Comparison between ARPES and DFT in Z plane ($k_z = \pi$) In figure S3, we can see that in addition to the experimental spectra taken in the $k_z = 0$ plane as shown in the main text, we also took limited spectra at the $k_z = \pi$ plane. In this figure, we can see good Fermi-surface agreement between experiment and DFT for the $x = 0.6$, and $x = 0.8$ compounds. By taking this into consideration along with the good agreement in the $k_z = 0$ plane, we argue that the DFT in the $k_z = \pi/2$ plane is likely to accurately reflect the electronic structure of the real material.

Experimental Lattice Parameters Used in DFT vs. Sr Substitution:

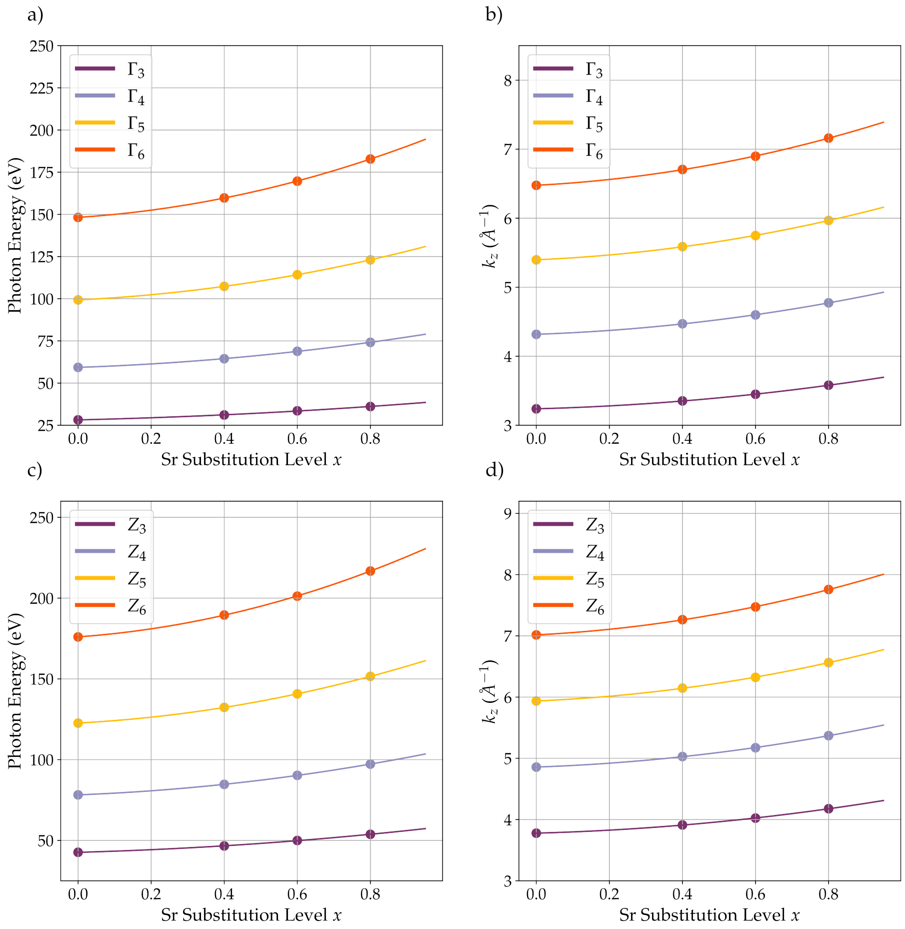

Figure S2: **$\mathbf{k_z}$ values and the associated photon energies required to reach them as a function of Sr-substitution in the tetragonal phase. a)** Γ points in photon energy as a function of Sr-substitution. Inner potentials used were 16 eV, 16 eV, 16 eV, and 17 eV for $x = 0$, $x = 0.4$, $x = 0.6$, and $x = 0.8$ compounds respectively. **b)** k_z values of associated Γ points. Points in k-space were calculated from experimental XRD data. **c)** Z points in photon energy as a function of Sr-substitution using the same inner potentials as **a)**. **d)** k_z values of associated Z points. Points were calculated in the same way as **b)**.

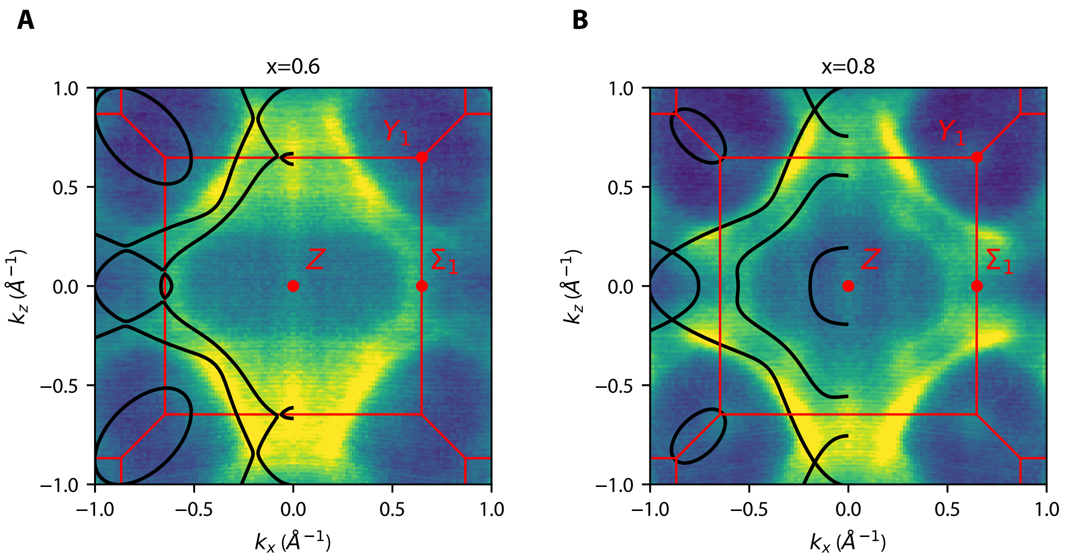

Figure S3: **ARPES Fermi-surfaces in the Z plane compared with DFT.** (**A**) $x = 0.6$ ARPES Fermi-surface taken at 91 eV, which corresponds to the $k_z = \pi$ or Z plane. DFT overlaid in black. (**B**) $x = 0.8$ ARPES Fermi-surface taken at 98 eV, which corresponds to the $k_z = \pi$ or Z plane. DFT overlaid in black. ARPES has been symmetrized along k_x in both maps to minimize intensity variation due to matrix element effects. Both maps were taken with LH (p) polarization.

Table S1: **Experimentally determined structures used in DFT calculations.** All structures listed are the respective primitive cells at each substitution level.

Compound	$BaNi_2As_2$	$Ba_{0.4}Sr_{0.6}Ni_2As_2$	$Ba_{0.25}Sr_{0.75}Ni_2As_2$	$Ba_{0.2}Sr_{0.8}Ni_2As_2$
Temperature (K)	250K	250K	250K	250K
Crystal System	BCT (I4/mmm)	BCT (I4/mmm)	BCT (I4/mmm)	BCT (I4/mmm)
Lattice Constants	a=4.1442, $\alpha = 108.53879°$<br>b=4.1442, $\beta = 108.53879°$<br>c=6.51713, $\gamma = 90°$	a=4.1442, $\alpha = 109.51935°$<br>b=4.1442, $\beta = 109.51935°$<br>c=6.201567, $\gamma = 90°$	a=4.1468, $\alpha = 109.84047°$<br>b=4.1468, $\beta = 109.84047°$<br>c=6.108972, $\gamma = 90°$	a=4.1525, $\alpha = 110.14687°$<br>b=4.1525, $\beta = 110.14687°$<br>c=6.028115, $\gamma = 90°$
Volume ($\AA^3$)	99.97472	93.86751	92.15740	90.77971
Wyckoff Positions	Ba (1a): 0,0,0<br>Ni (2d): 0.25,0.75,0.5<br>As (2e):<br>0.65261,0.65261,0.30522	Sr (1a): 0,0,0<br>Ni (2d): 0.25,0.75,0.5<br>As (2e):<br>0.64543,0.64543,0.29086	Sr (1a): 0,0,0<br>Ni (2d): 0.25,0.75,0.5<br>As (2e):<br>0.643,0.643,0.286	Sr (1a): 0,0,0<br>Ni (2d): 0.25,0.75,0.5<br>As (2e):<br>0.64082,0.64082,0.28164

In table S1 we show the experimentally refined structures used in our DFT calculations. For the parent $x = 0$ compound, Ba was used, while for the other substitution levels, Sr was fully substituted for Ba. As Sr is increased, we see an associated reduction in the cell volume, and change in the lattice constants. Interestingly, the **a** and **b** lattice constants do not shift appreciably until $x = 0.75$, where they increase sharply. For more information about the structural changes as Sr is substituted for Ba, including bond angle information, see ref. (5).

Atomic and Orbital Projections: In figure S4, we plot the contributions of each atom, Ba, Ni, and As, to the spectral weight on the Fermi surface of $Ba_{1-x}Sr_xNi_2As_2$. In figs. S4 a1)-a4), we plot the Fermi surface with the color at each point representing the largest contribution out of each of the three atoms. In figs. S4 b1)-b4), we resolve the angular dependence of the atomic contributions along the innermost P pocket, and in c1)-c3) as well as in d1)-d3), we do the same for the N pockets. We can see that for most of the pockets on the Fermi surface, Ni contributes the largest share of the spectral weight. For the innermost P pocket, the dominant contribution arises from As p states, but the Ni d states contribute a large fraction of the spectral weight ($\sim 45\%$). For the purposes of our analysis, we focus only on the Ni d-orbitals.

In figures S5 and S6, we see the Ni d-orbital projections of the main states along high symmetry directions. As is clearer in Fig. S6, the system shows strong hybridization, with all 5 Ni d-orbitals contributing to the spectral weight. In this orbital subspace, the main states at E_f have dominant $\mathrm{d_{xy}}$, $\mathrm{d_{xz}}$/$\mathrm{d_{yz}}$, and $\mathrm{d_{x^2-y^2}}$ orbital characters, as shown in Fig. S5.

In figure S7, we investigate the Ni d-orbital projections on the N and P pockets further, where we see that $\mathrm{d_{xz}}$, and $\mathrm{d_{yz}}$ states become more prominent at E_f as a function of Sr substitution. In figure S7 b1)-b4), the full angular-resolved Ni d-orbital composition of the innermost P electron pocket is shown as a function of Sr substitution. At the substitution level close to the Lifshitz transition, the $\mathrm{d_{xz}}$ and $\mathrm{d_{yz}}$ components become dominant at $0°$ and $90°$, which are the directions that point towards the N hole pockets. The Ni d-orbital content on the N hole

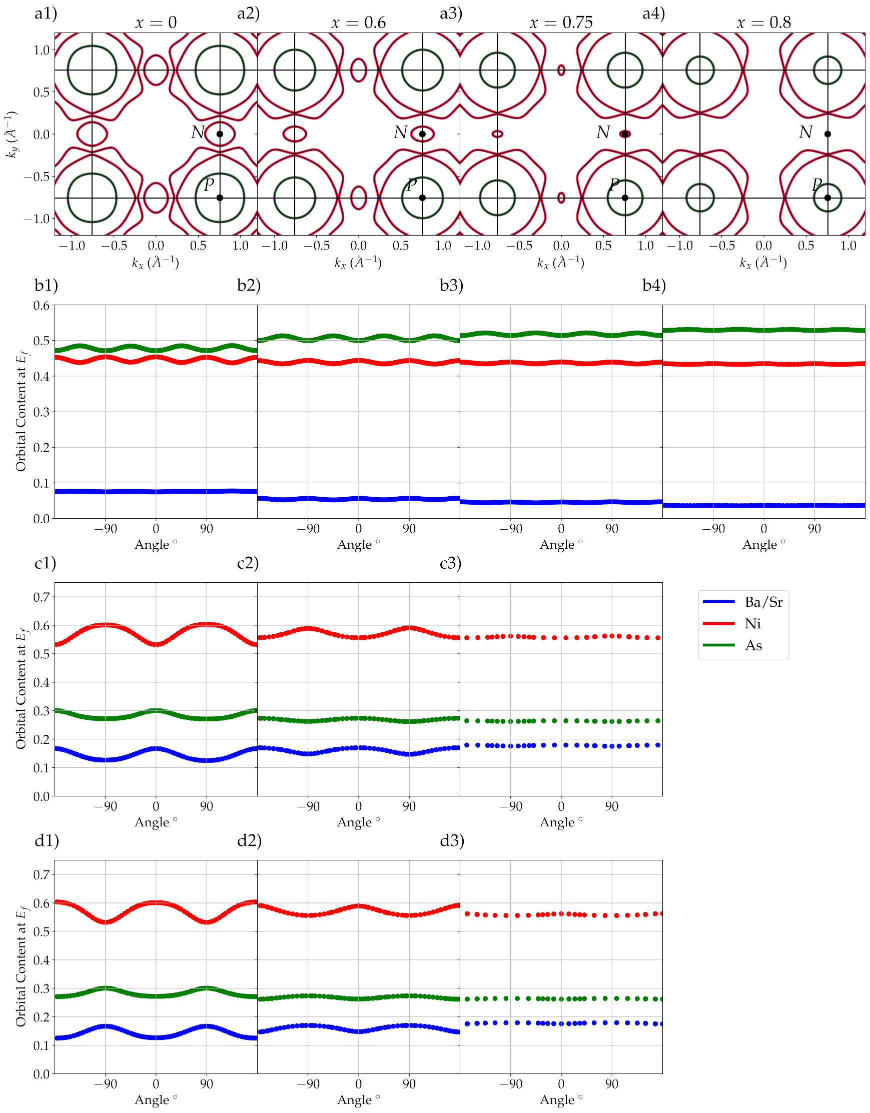

Figure S4: **Atomic projections at $\mathbf{E_f}$. a1)-a4)** N-P plane Fermi surfaces as a function of Sr substitution, showing the N pocket disappearing for $x > 0.75$. Color weights at each point correspond to the atom with the largest projection at that E, k point. **b1)-b4)** Angular dependence of the atomic projections at E_f on the innermost P electron pocket as a function of Sr substitution. Here, $0°$ corresponds to the $+k_x$ axis, and $90°$ corresponds to the $+k_y$ axis. **c1)-c3)** Angular dependence of the atomic projections at E_f on the N pocket at $k_x = 0$, $k_y = \pi$ as a function of Sr substitution. **d1)-d3)** Same as **c1)-c3)**, but for the other symmetry-related N pocket found at $k_x = \pi$, $k_y = 0$.

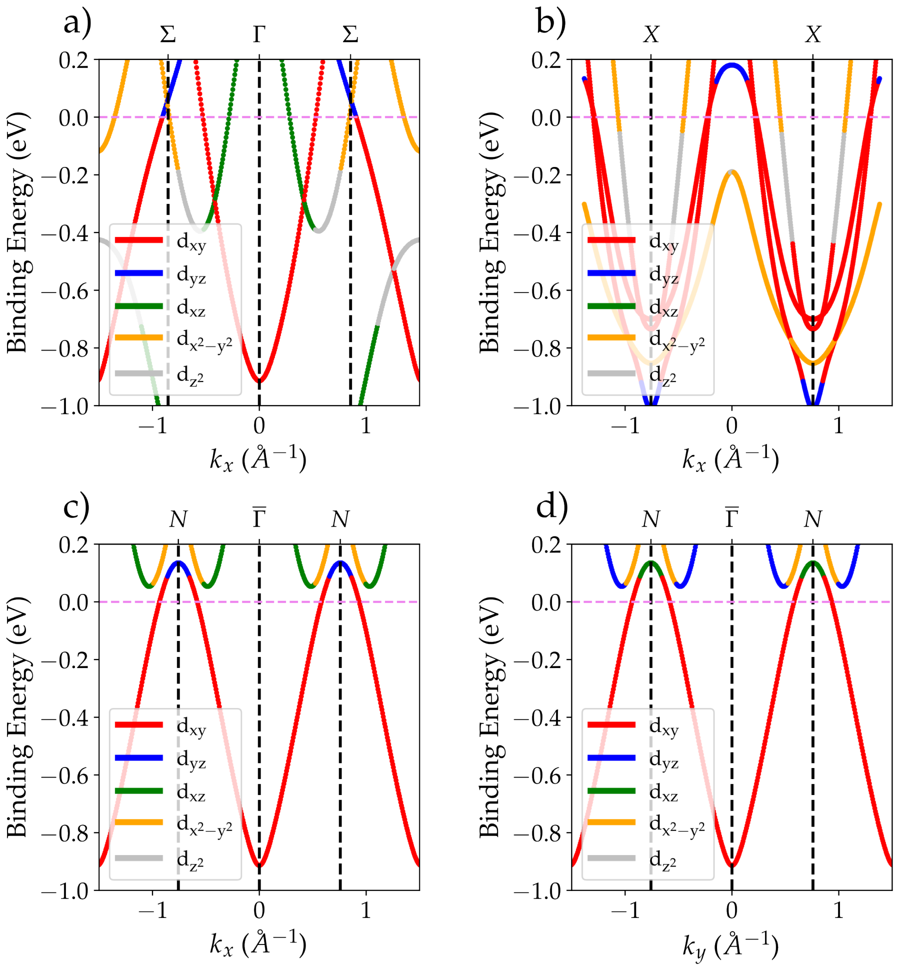

Figure S5: $\mathbf{E(k)}$ **dispersion for the** $\mathbf{x = 0}$ **parent compound with Ni d-orbital projections obtained from DFT.** Black dashed lines indicate high symmetry points. The color at each band point represents the orbital with the largest spectral weight at that E, k point. **a)** $\Sigma\Gamma\Sigma$ cut with orbital projection. The bands found between Γ and Σ are hybridized d_{z^2}, d_{xz} and $d_{x^2-y^2}$ states, while the band centered at Γ has majority d_{xy} character. **b)** X-X cut with orbital projection. The two outermost pockets centered at the X point have dominant d_{xy} orbital character, while the innermost pocket has hybridized d_{z^2} and $d_{x^2-y^2}$ orbital content. **c)** N-N Cut showing the hole pocket centered at the N point. This pocket has substantial d_{xy} character, but gains d_{yz} character at the band-top. **d)** Same cut as **c)**, but along the k_y direction, showing the greater d_{xz} orbital character at the band-top.

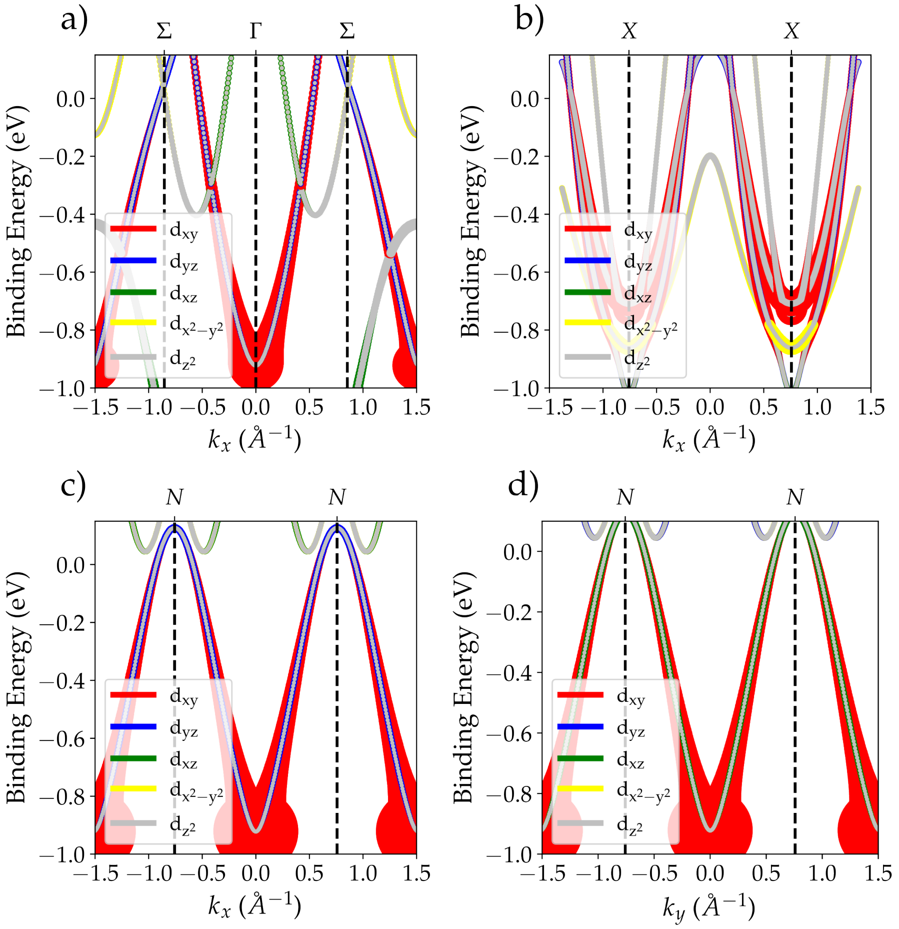

Figure S6: $\mathrm{E}(\mathbf{k})$ **dispersion for the** $\mathbf{x} = 0$ **parent compound with Ni d-orbital projections obtained from DFT, but with the Ni d-orbital projections visualized as line thickness.** Note the strong hybridization of orbitals near E_f.

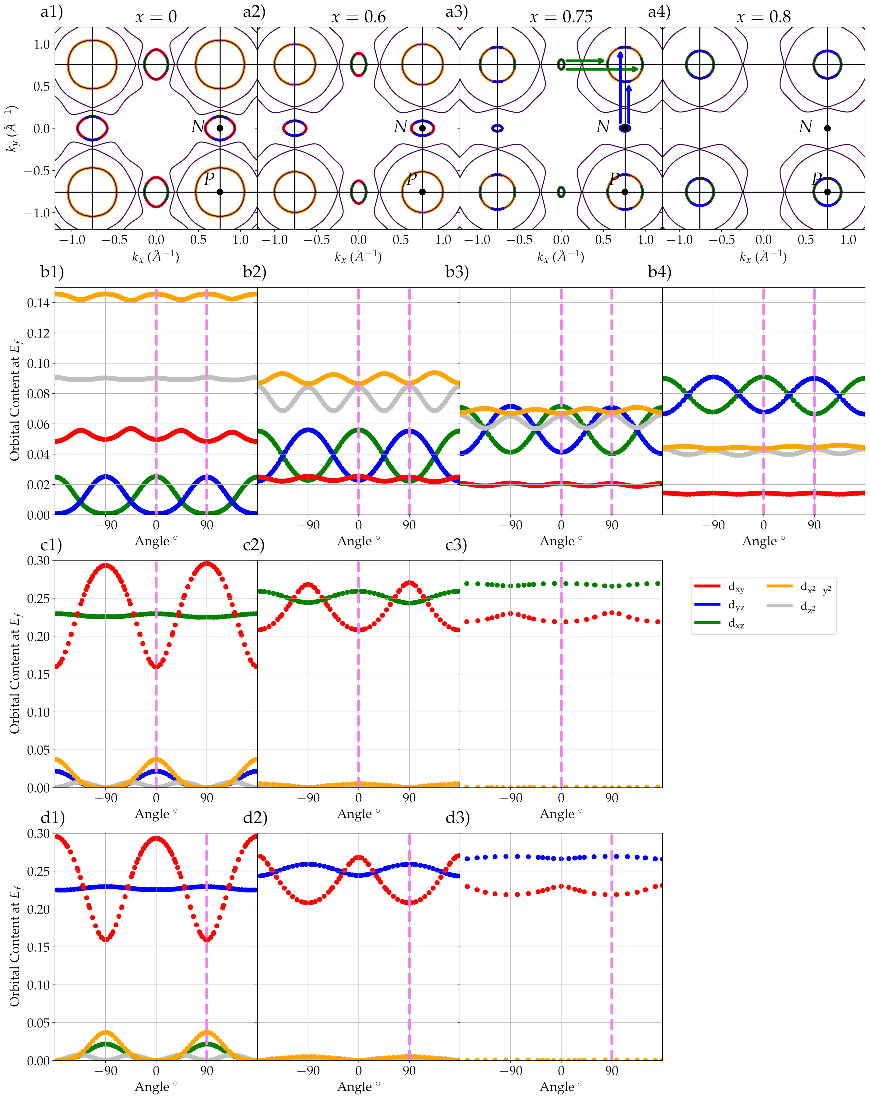

Figure S7: **Ni d-orbital projections at E_f. a1)-a4)** N-P plane Fermi surfaces as a function of Sr substitution, showing the N pocket disappearing for $x > 0.75$. Green and blue arrows in figure **a3)** show the k vectors that connect the d_{xz}, and d_{yz} states between the N and P pockets. **b1)-b4)** Angular dependence of the orbital projections at E_f on the innermost P electron pocket as a function of Sr substitution. Here, 0° corresponds to the $+k_x$ axis, and 90° corresponds to the $+k_y$ axis. Magenta lines correspond to the k vectors in panel **a3)**. **c1)-c3)** Angular dependence of the orbital projections at E_f on the N pocket at $k_x = 0$, $k_y = \pi$ as a function of Sr substitution. Magenta lines correspond to the k vectors connecting the N and P pockets along the $+k_x$ direction in panel **a3)**. **d1)-d3)** Same as **c1)-c3)**, but for the other symmetry-related N pocket found at $k_x = \pi$, $k_y = 0$.

pockets are shown in figures S7 c1)-c3), and d1)-d3). Similarly to the P pocket, the $\mathrm{d_{xz}}$, and $\mathrm{d_{yz}}$ orbitals become more dominant around the entire pocket as a function of Sr substitution. At the critical substitution level, the angles match with their respective counterparts on the P pocket. This Ni d-orbital nesting of the states at 0° and 90° is consistent with nematic fluctuations in the $\mathrm{B_{1g}}$ symmetry channel.
